# Neutrophil to lymphocyte ratio at diagnosis can estimate vasculitis activity and poor prognosis in patients with ANCA-associated vasculitis: a retrospective study

**DOI:** 10.1186/s12882-018-0992-4

**Published:** 2018-07-31

**Authors:** Sung Soo Ahn, Seung Min Jung, Jason Jungsik Song, Yong-Beom Park, Sang-Won Lee

**Affiliations:** 1 Division of Rheumatology, Department of Internal Medicine, Yonsei University College of Medicine, 50-1 Yonsei-ro, Seodaemun–gu, Seoul, 03722 Republic of Korea; 2 Institute for Immunology and Immunological Diseases, Yonsei University College of Medicine, Seoul, Republic of Korea

**Keywords:** Antineutrophil cytoplasmic antibody-associated vasculitis, Neutrophil to lymphocyte ratio, Vasculitis activity, Prognosis

## Abstract

**Background:**

Neutrophil to lymphocyte ratio (NLR) was introduced to predict poor prognosis in various diseases, but not all variants of ANCA-associated vasculitis (AAV). In this study, we aimed to investigate whether NLR at diagnosis can estimate vasculitis activity at diagnosis and poor prognosis during follow-up in patients with AAV.

**Methods:**

We retrospectively reviewed the medical records of 160 patients with AAV. We collected clinical and laboratory data at diagnosis and obtained remission and death as poor prognosis. We stratified AAV patients into three groups according to tertile and defined the lower limit of each highest tertile as the optimal cut-off (5.9 for NLR and 15.0 of Birmingham vasculitis activity score [BVAS] for severe AAV).

**Results:**

The mean age at diagnosis was 55.2 years and 48 patients were men. In the univariable linear regression analysis, BVAS was negatively correlated with lymphocyte count and positively correlated with erythrocyte sedimentation rate (ESR), C-reactive protein (CRP) and NLR. In the multivariable linear regression analyses of ESR and CRP with either lymphocyte count or NLR, lymphocyte count (β = − 0.160) and NLR (β = 0.169) were associated with BVAS. Patients having NLR ≥ 5.9 exhibited severe AAV more frequently than those having NLR < 5.9 at diagnosis (relative 2.189, *P* = 0.023). Patients having NLR ≥ 5.9 exhibited a higher frequency of AAV relapse, but not death, than those having NLR < 5.9 (*P* = 0.016).

**Conclusions:**

NLR at diagnosis can estimate vasculitis activity at diagnosis and predict relapse during follow-up in patients with AAV.

## Background

Antineutrophil cytoplasmic antibody (ANCA)-associated vasculitis (AAV) is a group of three systemic vasculitides involving small vessels from capillaries to intraparenchymal arterioles and venules: microscopic polyangiitis (MPA), granulomatosis with polyangiitis (GPA) and eosinophilic granulomatosis with polyangiitis (EGPA) [1]. MPA and GPA exhibit similar clinical manifestations of pulmonary, renal and ear-nose-throat manifestations, despite differences in genetic backgrounds, aetiologies, ANCA type, and histologic findings [1–3], whereas, EGPA shows both necrotising vasculitis and allergic components such as asthma and eosinophilia [1, 2, 4].

Neutrophil to lymphocyte ratio (NLR) has been recently introduced and widely used to predict poor prognosis in several cancers and inflammatory diseases [5, 6]. Neutrophil count may be often directly proportional to the inflammatory burdens, furthermore, activated neutrophils are very closely associated with the pathogenesis of AAV. By contrast, lymphocyte count may decrease in autoimmune inflammatory diseases [7]. Particularly, lymphopenia with low numbers of CD4+ T cells can be observed in GPA due to an extensive recruitment of peripheral T cells to the affected tissues [8]. Therefore, it can be reasonably speculated that NLR may reflect the inflammatory burdens in patients with systemic vasculitis. So far, NLR has been reported to be associated with disease activity and prognosis in Takayasu arteritis, Behcet disease, Kawasaki vasculitis and Henoch Schonlein purpura [9–11]. However, there were only a few reports the clinical role of NLR in patients with AAV [12, 13]. Furthermore, there was no study to demonstrate the association of NLR with both vasculitis activity and poor prognosis including relapse and death in a considerable number of patients with MPA, GPA and EGPA to date. Hence, in this study, we aimed to investigate whether NLR at diagnosis can estimate vasculitis activity at diagnosis and poor prognosis during follow-up in 160 patients with AAV, who were not administered immunosuppressive drugs before AAV diagnosis.

## Methods

### Patients

We retrospectively reviewed the medical records of 160 patients with AAV according the inclusion criteria as follows: i) patients who were first classified as AAV from October 2000 to September 2017 at the Department of Internal Medicine, Yonsei University College of Medicine, Severance Hospital, where this study was conducted as a monocentric investigation; ii) patients who fulfilled the American College of Rheumatology 1990 criteria for the classification for AAV and then reclassified by the 2007 European Medicines Agency algorithm modified by the 2012 revised Chapel Hill Consensus Conferences Nomenclature of Vasculitis [1–4]; iii) patients who had well-documented medical records with which to calculate items of Birmingham vasculitis activity score (BVAS) and five factor score (FFS (2009)) at diagnosis [14–16]; iv) patients who had results on perinuclear (P)-ANCA and cytoplasmic (C)-ANCA or myeloperoxidase (MPO)-ANCA and proteinase 3 (PR3)-ANCA at diagnosis [17]; v) patients who had no concomitant or previous medical conditions to disturb AAV classification, which was confirmed by the 10th revised International Classification of Diseases [18]; and vi) patients who received no immunosuppressive drugs prior to diagnosis of AAV, which was searched by the Korean Drug Utilization Review system. This study was approved by the Institutional Review Board of Severance Hospital (4–2017-0673), who waived the need for patient written informed consent, as this was a retrospective study.

### Clinical data

We obtained age and gender as demographic data at the time of the first diagnosis of AAV and searched the initial ANCAs. When an AAV patient exhibited an item described in BVAS, we considered him or her to have an organ-specific involvement of AAV, regardless of tissue biopsy findings as below: general manifestation including muscle pain, joint symptoms, fever and weight loss ≥2 kg; cutaneous manifestation including skin rashes and ulcerations; mucous membrane / eyes manifestation including oral or genital ulceration, inflammation in sclera or conjunctiva, impairment in visual function, uveitis and retinitis; ear nose throat manifestation including inflammation in nasal passage or paranasal sinus and hearing loss; chest manifestation including inflammation in both lung parenchyma and pleura; cardiovascular manifestation including coronary arterial occlusion, heart failure and pericarditis; abdominal manifestation including gastrointestinal bleeding and mesenteric arterial occlusion; renal manifestation including proteinuria > 1+ on urine stick, haematuria ≥10 RBCs/HPF and renal dysfunction; nervous system manifestation including central and peripheral neuropathies. We also calculated the total score of BVAS and FFS (2009) at diagnosis.

### Laboratory data

We collected laboratory results at diagnosis, which represent the inflammatory burdens in the real clinical settings including complete blood counts [white blood cell (WBC), neutrophil, lymphocyte, and platelet counts]; erythrocyte sedimentation rate (ESR) and C-reactive protein (CRP) before the administration of immunosuppressive drugs to AAV patients. The follow-up duration was defined as the duration from diagnosis to the last visit in patients without relapse, whereas it was defined as the time from diagnosis to the first relapse in patients with relapse. Therefore, the follow-up duration in this study meant the relapse free period of AAV.

### Prognosis

Remission was determined as no active disease requiring the maintenance therapy, relapse was defined as active disease after remission [19]. We also counted all cause death in AAV patients.

### Equations of NLR and optimal cut-off

NLR was calculated as a ratio of neutrophil count over lymphocyte count at diagnosis [NLR = neutrophil count (/uL) / lymphocyte count (/uL)] [9, 10]. We stratified AAV patients into three groups according to tertile and define the lower limit of each highest tertile as the optimal cut-off [6]. The optimal cut-offs were set at 15.0 for severe AAV based on BVAS and 5.9 for NLR. In particular, in this study, we discretionally define severe AAV when BVAS is 15.0 or greater.

### Statistical analyses

We expressed continuous variables as a mean ± standard deviation, and categorical variables as number (%). We assessed the standardised correlation coefficient by the multivariable linear regression analysis using variables with significance in the univariable analysis. We compared categorical variables between the two groups, and analysed the relative risk (RR) using the chi square and Fisher’s exact tests. Also we compared cumulative relapse free and patient survivals between the two groups using the Kaplan-Meier survival analysis. We conducted all statistical analyses using SPSS software (version 23 for windows; IBM Corp., Armonk, NY, USA). *P*-values < 0.05 were considered statistically significant.

## Results

### Baseline characteristics of 160 patients with AAV

The baseline characteristics are described in Table 1. Eight-five of 160 patients (53.1%) were classified as MPA, 41 patients (25.6%) as GPA and 34 patients (21.3%) as EGPA. The mean age at diagnosis was 55.2 years and 48 patients (30.0%) were men. Ninety-nine patients (61.9%) and 27 patients (16.9%) had MPO-ANCA (or P-ANCA) and PR3-ANCA (or C-ANCA), respectively. Seven patients (4.4%) had MPO-ANCA (or P-ANCA) as well as PR3-ANCA (or C-ANCA), and 41 patients (25.6%) had no ANCA. The most common clinical manifestation of AAV at diagnosis was renal manifestation (59.4%), followed by chest (52.5%) and general (44.4%) manifestations. The mean BVAS at diagnosis was 11.9 and the mean FFS (2009) at diagnosis was 1.3. In terms of laboratory results related to the inflammatory burdens at diagnosis, the mean WBC, neutrophil, lymphocyte and platelet counts were 10,175.6/mm^3^, 7227.5/mm^3^, 1564.0/mm^3^ and 327,500.0/mm^3^, respectively. The mean NLR was 6.6. The mean follow-up duration was 55.6 months. During the follow-up of more than 12 weeks, 43 patients (26.9%) exhibited relapse after remission and 14 patients (8.8%) died.

**Table 1:** Baseline characteristics of 160 patients with AAV

Variables	Values
Variants of AAV	
MPA	85 (53.1)
GPA	41 (25.6)
EGPA	34 (21.3)
Demographic data at diagnosis	
Age (year old)	55.2 ± 15.1
Male gender (*N*, (%))	48 (30.0)
ANCA at diagnosis (*N*, (%))	
MPO-ANCA (or P-ANCA)	99 (61.9)
PR3-ANCA (or C-ANCA)	27 (16.9)
MPO-ANCA (or P-ANCA) and PR3-ANCA (or C-ANCA)	7 (4.4)
ANCA negative	41 (25.6)
Clinical manifestations at diagnosis (*N*, (%))	
General	71 (44.4)
Cutaneous	37 (23.1)
Mucous membranes/eyes	12 (7.5)
Ear Nose Throat (ENT)	56 (35.0)
Chest	84 (52.5)
Cardiovascular	45 (28.1)
Abdominal	10 (6.3)
Renal	95 (59.4)
Nervous system	52 (32.5)
Vasculitis activity and prognostic factors at diagnosis	
BVAS or BVAS for GPA	11.9 ± 7.6
FFS (2009)	1.3 ± 1.0
Laboratory results at diagnosis	
WBC (/mm^3^)	10,175.6 ± 4758.2
Neutrophil (/mm^3^)	7227.5 ± 4047.2
Lymphocyte (/mm^3^)	1564.0 ± 721.2
Platelet (×1,000/mm^3^)	327.5 ± 141.9
ESR (mm/hr)	60.1 ± 37.4
CRP (mg/L)	43.0 ± 56.5
NLR at diagnosis	6.6 ± 8.3
Prognosis	
Follow-up duration (months)	55.6 ± 51.5
Relapse (*N*, (%))	43 (26.9)
Death (*N*, (%))	14 (8.8)

Values are expressed as mean and standard deviation or *N* (%)

*AAV* antineutrophil associated vasculitis, *MPA* microscopic polyangiitis, *GPA* granulomatosis with polyangiitis, *EGPA* eosinophilic granulomatosis with polyangiitis, *MPO* myeloperoxidase, *ANCA* antineutrophil cytoplasmic antibody, *P-ANCA* perinuclear ANCA, *PR3* proteinase 3, *C-ANCA* cytoplasmic ANCA, *BVAS* Birmingham vasculitis activity score, *FFS* five factor score, *WBC* white blood cell, *ESR* erythrocyte sedimentation rate, *CRP* C-reactive protein, *NLR* neutrophil to lymphocyte ratio

### Univariable and multivariable linear regression analyses

In the univariable linear regression analysis, BVAS was negatively correlated with lymphocyte count (*r* = − 0.198, *P* = 0.012) and positively correlated with ESR (*r* = 0.218, *P* = 0.006) and CRP (*r* = 0.169, *P* = 0.033). BVAS was also significantly correlated with NLR (*r* = 0.204, *P* = 0.009) (Table 2). We performed the multivariable linear regression analyses of ESR and CRP with either lymphocyte count or NLR. In terms of multivariable linear regression analysis of lymphocyte, ESR and CRP, only lymphocyte count was significantly associated with BVAS (β = − 0.160, 95% confidence interval [CI] -0.003, 0.000, *P* = 0.045). In terms of multivariable linear regression analysis of NLR, ESR and CRP (*R* = 0.279), only NLR was significantly associated with BVAS (β = 0.169, 95% CI 0.010, 0.299, *P* = 0.036) (Table 2).

**Table 2:** Univariable and multivariable linear regression analyses of BVAS and variables related to the inflammatory burdens in 160 patients with AAV

	Univariable analysis			Multivariable analysis (ESR, CRP and Lymphocyte)			Multivariable analysis (ESR, CRP and NLR)		
	Regression Coefficient (Crude B)	Correlation Coefficient (R = β)	*P* value	Standardized β^*^	95% confidence interval	*P* value	Standardized β^*^	95% confidence interval	*P* value
Demographic data at diagnosis									
Age (years old)	0.027	0.053	0.504						
Laboratory data at diagnosis									
WBC (/mm^3^)	0.000	0.095	0.232						
Neutrophil (/mm^3^)	0.000	0.114	0.150						
Lymphocyte (/mm^3^)^*^	− 0.002	−0.198	0.012	−0.160	− 0.003, 0.000	0.045	N/A	N/A	N/A
Platelet (×1,000/mm^3^)	0.003	0.060	0.449						
ESR (mm/hr)	0.044	0.218	0.006	0.167	−0.003, 0.071	0.074	0.177	−0.001, 0.073	0.058
CRP (mg/L)	0.023	0.169	0.033	0.044	−0.019, 0.031	0.644	0.029	−0.021, 0.029	0.757
NLR	0.187	0.204	0.009	N/A	N/A	N/A	0.169	0.010, 0.299	0.036

^*^We performed multivariable linear regression analyses of ESR and CRP with either lymphocyte count or NLR

*BVAS* Birmingham vasculitis activity score, *AAV* antineutrophil associated vasculitis, *ESR* erythrocyte sedimentation rate, *CRP* C-reactive protein, *WBC* white blood cell, *NLR* neutrophil to lymphocyte ratio

### RR of severe AAV based on BVAS

**Fig. 1:**
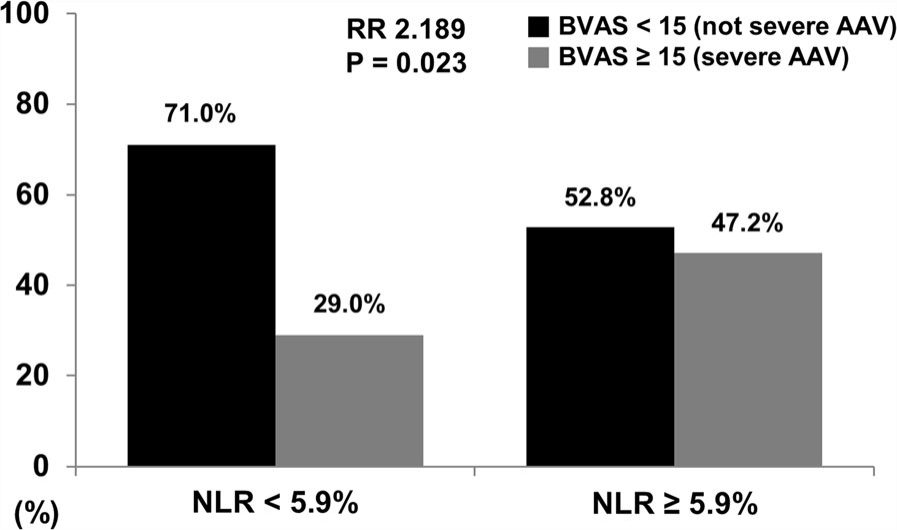
Relative risk of severe AAV based on BVAS. Patients having NLR ≥ 5.9 exhibited the higher frequency of severe AAV than those having NLR < 5.9 (47.2% vs. 29.0%, RR 2.189, *P* = 0.023). AAV; ANCA-associated vasculitis: ANCA: antineutrophil cytoplasmic antibody; BVAS: Birmingham vasculitis activity score; NLR: neutrophil to lymphocyte ratio

When we classified AAV patients into two groups based on the cut-off of NLR, patients having NLR ≥ 5.9 exhibited the higher frequency of severe AAV than those having NLR < 5.9 (47.2% vs. 29.0%, *P* = 0.023). Particularly, patients having NLR ≥ 5.9 had a significantly higher risk of severe AAV than those not having (RR 2.189, 95% CI 1.107, 4.330) (Fig. 1).

### A predictor of relapse and death during follow-up

**Fig. 2:**
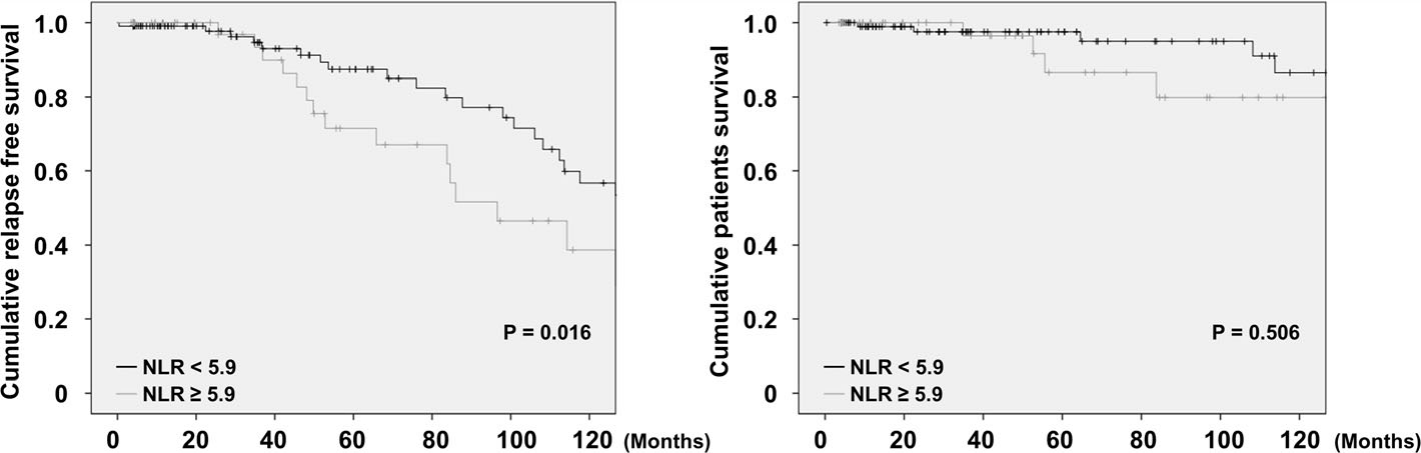
A predictor of relapse of AAV. Patients having NLR ≥ 5.9 exhibited the higher frequency of relapse of AAV than those having NLR < 5.9 (*P* = 0.016). AAV; ANCA-associated vasculitis: ANCA: antineutrophil cytoplasmic antibody; NLR: neutrophil to lymphocyte ratio

We evaluated whether the highest tertile of NLR (5.9 or greater) at diagnosis can predict relapse of AAV and death during the follow-up using Kaplan-Meier survival analysis. Cumulative relapse free and patient survival rates were depicted in Fig. 2. Patients having NLR ≥ 5.9 exhibited the higher frequency of relapse of AAV than those having NLR < 5.9 (*P* = 0.016). Thus, NLR has a potential of a predictor of relapse of AAV during the follow-up. However, there was no significant difference in cumulative patient survival rate between patients having NLR ≥ 5.9 and those NLR < 5.9 at diagnosis.

## Discussion

In this study, we investigated whether NLR at diagnosis can estimate vasculitis activity at diagnosis and poor prognosis during follow-up in 160 patients with AAV in a single centre. First, in terms of vasculitis activity of AAV, we conclude that lymphocyte count and NLR are significantly correlated with BVAS, comparable to ESR and CRP. Meanwhile, among four variables, lymphocyte count and NLR are significantly associated with BVAS. The statistical significance of the association between BVAS and NLR was slightly higher than that between BVAS and lymphocyte count (β = 0.169 vs. β = − 0.160). In addition, NLR ≥ 5.9 (RR 2.189) can estimate severe AAV based on BVAS. Second, in terms of prognosis of AAV, we conclude that NLR ≥ 5.9 at diagnosis is a predictor of relapse of AAV, but not death, during follow-up. Therefore, we believe that NLR at diagnosis is a useful marker to estimate vasculitis activity at diagnosis and poor prognosis during follow-up in AAV patients.

In addition to NLR, lymphocyte count also exhibited a significant correlation and association with BVAS along with ESR and CRP. Unlike NLR, lymphocyte count is automatically counted and reported, suggesting that lymphocyte count is much more convenient than NLR. Nonetheless, NLR has been widely proposed to estimate the inflammatory burdens and predict prognosis than lymphocyte count in various diseaes. NLR includes two different lineages of immune cells, neutrophils and lymphocytes. Neutrophils are mainly in charge of nonspecific and early systemic inflammation. Neutrophil count may be elevated by infections or temporarily by glucocorticoid use, whereas it may be reduced by neutrophil-consuming medical conditions or immunosuppressive drugs. Meanwhile, lymphocyte participates in relatively late immune reactions. Lymphocyte count may also be affected by general health and stress or various autoimmune diseases. Therefore, NLR, in which two lineages of immune cells possessing different characters are integrated, is considered a more reliable and complementary marker than counts of single immune cells such as lymphocytes [4, 20].

In this study, we first demonstrated that NLR ≥ 5.9 at diagnosis can predict relapse of AAV during follow-up. Calculating NLR at diagnosis of AAV implies that they mainly reflect the vasculitis activity of AAV before the administration of immunosuppressive drugs. In our previous studies, we demonstrated that BVAS at diagnosis representing the initial inflammatory burdens could predict poor prognosis such as relapse or refractory disease in patients with AAV [21, 22]. Therefore, the clinical role of NLR at diagnosis to predict relapse of AAV can be explained by the positive link between NLR and BVAS. With these results, we suggest that physicians may calculate NLR at diagnosis to predict poor prognosis of AAV during follow-up. Furthermore, we also suggest that the more frequent visits, laboratory tests and evaluation of treatment efficacy may be necessary in AAV patients having NLR ≥ 5.9.

The number of neutrophils can be increased by the use of glucocorticoids, whereas it can be decreased by the use of immunosuppressive drugs by bone marrow suppression. Therefore, we included only patients who had neither glucocorticoid nor immunosuppressive drugs in this study. In addition, we excluded 12 patients with ischaemic heart disease or peripheral vascular diseases, as NLR might be influenced by atherosclerosis or peripheral vascular diseases [23, 24].

Our study has two advantages. First, we demonstrated the clinical roles of NLR at diagnosis to not only estimate vasculitis activity at diagnosis, but also predict relapse during follow-up in patients with all variants of AAV. Second, we could control the clinical and laboratory confounding factors including inter-observer or inter-centric variation due to a single-centric study. However, this study also has several limitations. First, we could not perform the subgroup analysis of clinical features of patients with NLR ≥ 5.9, but without severe AAV or relapse due to the retrospective study-design. Second, the number of AAV patients in this study was not large enough to represent the ethnic feature of Korean patients with AAV. If future studies with the larger number of patients can calculate BVAS and NLR prospectively, they might reveal the reliable and valuable results of NLR to not only estimate the current BVAS, but also predict poor prognosis during follow-up.

## Conclusions

NLR at diagnosis can estimate vasculitis activity at diagnosis and predict relapse during the follow-up in patients with AAV. Thus, we suggest that physicians should pay more attention to patients with NRL at diagnosis ≥5.9, encourage them to visit more often and prolong the period of maintenance therapy even in those achieving remission.

## Abbreviations

AAV: ANCA-associated vasculitis
ANCA: Antineutrophil cytoplasmic antibody
BVAS: Birmingham vasculitis activity score
C: Cytoplasmic
CI: Confidence interval
CRP: C-reactive protein
EGPA: Eosinophilic granulomatosis with polyangiitis
ESR: Erythrocyte sedimentation rate
FFS: Five factor score
GPA: Granulomatosis with polyangiitis
MPA: Microscopic polyangiitis
MPO: Myeloperoxidase
NLR: Neutrophil to lymphocyte ratio
P: Perinuclear
PR3: Proteinase 3
RR: Relative risk
WBC: White blood cell
